# On Becoming a Senior Staff Nurse in Taiwan: A Narrative Study

**DOI:** 10.3390/healthcare13151896

**Published:** 2025-08-04

**Authors:** Yu-Jen Hsieh, Yu-Tzu Dai

**Affiliations:** School of Nursing, College of Medicine, National Taiwan University, Taipei 100, Taiwan; yutzu@ntu.edu.tw

**Keywords:** narrative inquiry, professional identity, emotional labor, senior nurses, Confucian culture, cultural caregiving, relational resilience, gendered expectations, long-term commitment, well-being

## Abstract

**Background/Objectives**: Senior nurses in Taiwan shoulder layered responsibilities shaped by professional roles, gendered expectations, and family duty. Although Taiwan faces a persistent shortage of experienced clinical nurses, limited research has explored how long-serving nurses sustain identity and commitment across decades of caregiving. This study examines how senior staff nurses understand their journeys of becoming—and remaining—nurses within a culturally and emotionally complex landscape. **Methods**: Interviews were conducted between May 2019 and September 2023 in locations chosen by participants, with most sessions face-to-face and others undertaken via video conferencing during COVID-19. This narrative inquiry involved in-depth, multi-session interviews with five female senior staff nurses born in the 1970s to early 1980s. Each participant reflected on her life and career, supported by co-constructed “nursing life lines.” Thematic narrative analysis was conducted using McCormack’s five-lens framework and Riessman’s model, with ethical rigor ensured through reflexive journaling and participant validation. **Results**: Three overarching themes emerged: (1) inner strength and endurance, highlighting silent resilience and the ethical weight of caregiving; (2) support and responsibility in relationships, revealing the influence of family, faith, and relational duty; and (3) role navigation and professional identity, showing how nurses revisit meaning, self-understanding, and tensions across time. Participants described emotionally powerful moments, identity re-connection, and cultural values that shaped their paths. **Conclusions**: These narratives offer a relational and culturally embedded understanding of what it means to sustain a career in nursing. Narrative inquiry created space for reflection, meaning-making, and voice in a system where such voices are often unheard. Identity was not static—it was lived, reshaped, and held in story.

## 1. Introduction

Healthcare workers are the backbone of the healthcare system; without them, the delivery of health services would not be possible. Among these professionals, nurses comprise the majority, accounting for nearly 60% of the workforce [1,2]. The issue of nursing shortages is not new. Since 2006, the WHO and International Council of Nurses (ICN) have raised concerns to garner global attention [3,4]. However, the situation has worsened, especially after the COVID-19 pandemic, and calls for more immediate and sustained attention [5,6].

Taiwan, like many other countries, faces an ongoing nursing shortage, driven by demographic changes and evolving healthcare demands. This shortage has been linked to the nation’s rapidly aging population, the increasing need for chronic disease management, and the retirement of the baby boomer generation [7]. In 2012, data showed that Taiwan’s average nurse age was only 29, significantly younger than counterparts in Hong Kong (46), Malaysia (40.6), and Thailand (37.8). In addition, the average years of nursing experience in Taiwan was just 6 to 7 years, compared with 38 years in Singapore, highlighting a lack of senior staff nurses with long-term clinical experience [8]. Although these data date back more than a decade, recent statistics indicate that little has changed. As of 2023, hospital nurse vacancy rates had risen from 4.5% to 6.5% post-COVID, and nurses with over ten years of experience continue to be the most likely to leave the profession. Those who remain in practice are often early career nurses or clinical novices [9].

Recent studies examining nursing workforce dynamics in Taiwan have predominantly employed quantitative approaches, exploring retention intention, stress coping, and structural predictors through large-scale survey designs [10,11,12,13,14,15,16]. While these studies offer valuable system-level insight, they rarely attend to how nurses themselves make sense of long-term professional commitment. Within the past five years, qualitative studies have remained scarce and primarily centered on pandemic-related challenges [17].

This persistent gap underscores a pressing need to understand how nurses in Taiwan progress toward senior roles. Examining their career paths can provide valuable insight into the factors that support or hinder long-term retention and professional development. Such understanding is critical to building a more stable, resilient nursing workforce, capable of meeting the demands of an aging society.

The aim of this study is to explore the experiences of senior staff nurses in becoming and remaining nurses in Taiwan. These trajectories are not shaped solely by institutional dynamics, but unfold within cultural expectations—including relational duty, gendered caregiving roles, and quiet endurance. While not the central focus of this study, such values offer important background for understanding how senior staff nurses in Taiwan make sense of their long-term professional experience. Specifically, the study seeks to answer the following research questions: What are the lived experiences of senior staff nurses throughout their careers? What life events hold particular meaning for them? What are the most memorable and impactful experiences in their clinical practice? Finally, what key factors and challenges have influenced their long-term commitment to the nursing profession?

Creswell and Creswell [18] identify five commonly used qualitative approaches: ethnography, grounded theory, phenomenology, case study, and narrative inquiry. Each method reflects distinct epistemological and analytical aims. This study adopted narrative inquiry not only as a research design but also as an ontological and epistemological stance. In contrast to grounded theory, which seeks to develop conceptual categories, and phenomenography, which aims to identify variations in experience across populations, narrative inquiry attends to lived experience as storied, relational, and culturally embedded. Its focus on temporality, voice, and co-construction makes it especially suitable for exploring how long-serving nurses in Taiwan sustain meaning, identity, and ethical commitment within layered professional and personal contexts.

In choosing narrative inquiry, we align with McCormack’s view that stories offer ethically situated windows into experience, and Riessman’s emphasis on how meaning arises through language, context, and dialogue. This approach enabled us to engage not only with what nurses experienced, but how they remembered, interpreted, and positioned those experiences within broader cultural narratives—particularly those shaped by duty, endurance, and caregiving across generations.

Thus, narrative inquiry was deemed the most appropriate approach to honor both the aims and ontological commitments of this research.

## 2. Materials and Methods

### 2.1. Research Design

This study adopted a narrative inquiry design to explore the experiences of senior staff nurses in becoming and remaining nurses in Taiwan. Narrative inquiry places participants at the center of the research process, allowing them to guide the inquiry through unstructured, in-depth interviews [19,20]. This approach helps the researcher gain insight into the lived experiences of senior staff nurses and the social phenomena surrounding them. Through storytelling, individuals connect their personal experiences to broader social contexts and give them meaning. This relational ontology forms the philosophical foundation of narrative inquiry, emphasizing that human life is shaped and understood through interactions with others and society [20,21]. The first author had previously completed a doctoral study in psychology at Fu Jen Catholic University using narrative inquiry, which deeply informed the ontological and ethical stance of the current research. This prior work emphasized self-narrative as a form of existential reflection and emotional healing, providing a foundational lens through which the present study’s relational and culturally embedded focus was developed [22]. Both the first author and the supervising professor are female, which supported empathetic engagement and gendered understanding across caregiving narratives.

In this study, narrative inquiry is regarded as both a methodology and a way of thinking about experience. Participants’ stories are examined across three dimensions of narrative space: temporality (past, present, and future), place, and sociality [20]. Their experiences are understood as holistic and deeply embedded in daily life, shaped by relationships, culture, and personal history.

This approach also echoes Bruner’s view that people construct reality through storytelling [23], and Carr’s idea that individuals make sense of their lives through temporal continuity and meaningful events [24]. Additionally, narrative inquiry is grounded in a hermeneutic tradition, drawing on Gadamer’s emphasis on interpretation as socially and historically situated [25]. Rather than conflicting with relational ontology, Gadamer’s philosophy complements it by underscoring that understanding emerges through relational and historically situated dialogue.

In the Taiwanese context, cultural values such as relational harmony, duty, and caregiving deeply influence both personal identity and professional life [26]. As this study is situated within that social context, narrative inquiry offers a meaningful way to explore how such cultural values shape the lived experiences of senior staff nurses. By engaging with participants’ stories, this study not only attends to their individual career journeys but also examines how social relationships and cultural expectations inform their long-term commitment to nursing.

### 2.2. Participants

This study employed purposive sampling to recruit senior staff nurses currently practicing in clinical settings across Taiwan. Inclusion criteria required participants to (1) have at least ten years of professional nursing experience, (2) have worked in clinical settings in Taiwan, and (3) be willing to participate in the study. Additionally, participants must have entered the nursing profession no later than 2005, ensuring that their education or early clinical practice overlapped with Taiwan’s 2003 SARS outbreak—a historically significant event that shaped their professional trajectories. This criterion was based on the assumption that nurses who had directly encountered the potential risks of public health crises, yet continued to serve on the front lines, likely found personal and professional meaning in their work. Their experiences offered a valuable opportunity to explore the deeper sources of long-term professional commitment. A total of five senior staff nurses participated in the study. Recruitment concluded once data saturation was reached—that is, when no new themes or significant insights were emerging from the interviews.

To deepen the methodological justification for this sampling strategy, we note the following: To further clarify the sampling rationale and demonstrate methodological rigor, this study intentionally employed a small, purposive sample in alignment with narrative inquiry’s emphasis on depth, temporality, and relational co-construction. Participants were selected not for statistical representativeness, but for the richness and complexity of their lived experience, shaped by decades of caregiving within Taiwan’s gendered and culturally embedded healthcare system.

Rigor was upheld through multi-session interviews, co-constructed lifeline mapping, member checking, and sustained reflexivity across sessions. Analytical credibility was further supported using McCormack’s five-lens framework and Riessman’s thematic narrative approach, enabling nuanced interpretation across emotional, contextual, and storied dimensions.

Rather than aiming for generalizability, this study embraces transferability by providing thick description, cultural specificity, and dialogical openness—allowing readers to assess resonance across parallel settings in nursing, culture, and identity work.

### 2.3. Ethical Considerations

Ethical approval for this study was obtained from the ethics committee of National Taiwan University Hospital (approval no. 201809088RINA). All participants were fully informed of their rights before participation. They were assured that their identities would remain anonymous, that all data collected would be kept confidential, and that they could withdraw from the study at any time without consequence. Written informed consent was obtained after the research purpose and procedures were clearly explained.

Interview recordings were de-identified and labeled with participant codes. These files were stored securely on the primary researcher’s password-protected computer. The data were used solely for research and educational purposes.

Although the researcher served as a co-author in co-constructing and presenting participants’ stories, the intellectual ownership and narrative rights remain with the participants themselves.

### 2.4. Data Collection

The first author, having clinical experience and prior engagement with the nursing community, directly invited participants to join the study. Participants were initially approached via professional networks and invited individually through LINE or email, depending on preference. Data were collected from May 2019 to September 2023 through in-depth, multiple-session interviews with five senior staff nurses. Participants were invited to choose a setting where they felt most comfortable. While most interviews were conducted face-to-face, video conferencing was used when necessary during the COVID-19 pandemic. All interviews were audio recorded with the participants’ prior informed consent.

Each participant was interviewed three to four times, with each session lasting approximately 90 to 120 min. During the first meeting, the researcher explained the study’s purpose and procedures and shared her professional background to foster rapport. Background information—including participants’ educational histories, clinical experiences, and family contexts—was collected during this session. No pilot testing was conducted. Instead, interviews followed an emergent and dialogical flow aligned with narrative inquiry principles, allowing participants to shape the narrative content through shared meaning-making. No invited participants declined or withdrew from the study; all five approached nurses consented to participate and completed the full series of interviews.

A central feature of the data collection process was the co-construction of a “nursing life line”—a visual timeline used to map key personal, professional, and historical milestones. Participants were invited to recall and reflect on events such as entering nursing school, encountering the 2003 SARS outbreak, experiencing career transitions, and navigating family responsibilities. The life line served not only as a memory aid but also as a narrative scaffold, helping participants situate their stories over time and uncover deeper meanings.

After the initial interview, the researcher used draw.io software v28.0.6 to create a digital version of each participant’s nursing life line. These visual maps were then sent to participants for review, clarification, or correction prior to subsequent interviews. This iterative exchange deepened the dialogic relationship and supported shared meaning-making across sessions.

Following each interview, the researcher wrote field notes and reflective journal entries to document contextual impressions and interpretive insights. Participants were also encouraged to share further thoughts or reflections between interviews via email or the LINE messaging app. Interviews continued until participants felt they had nothing more to share.

During the narrative construction process, the researcher drafted a written story for each participant based on interview transcripts, field notes and a reflective journal. These drafts were returned to participants for feedback and revision until they were comfortable with how their story was presented. Participants remained welcome to contact the researcher at any point should they wish to contribute additional stories or reflections.

### 2.5. Data Analysis

The analysis was conducted in two stages. In the first stage, the researcher worked closely with each participant’s transcript to develop an interpretive story. This process was guided by McCormack’s five-lenses, active listening, narrative processes, language, context, and moments [27,28], to support attention to emotional depth and contextual meaning. These stories were not summaries, but thoughtfully constructed narratives that captured the tensions, turning points, and values expressed by participants.

Influenced by Gadamer’s concept of the fusion of horizons [25], the researcher did not treat the transcripts as objective data. Instead, she engaged in reflective dialogue between the participants’ stories and her own understanding as a nurse-researcher. Through this interpretive process, the narratives became co-constructed and shaped by mutual resonance, rather than imposed categories.

Each interpretive story was returned to the respective participant for member checking. This process allowed participants to clarify meanings, affirm representations, or suggest revisions. This dialogical exchange ensured that the interpretations remained grounded in participants’ perspectives.

In the second stage, a thematic narrative analysis was conducted, following Riessman’s model [29]. The researcher focused on identifying what was storied across narratives by reading each interpretive story holistically rather than coding isolated excerpts. Recurring emotional threads and common situations participants faced, such as workplace bullying, moral tension, and emotional labor, were traced across stories. This approach preserved the coherence of each narrative while allowing for meaningful cross-case pattern recognition.

Although the interpretive stories served as the primary unit of analysis, they are not presented in full due to space limitations. However, all thematic insights were developed directly from these narratives, retaining the complexity and emotional nuance of participants’ experiences. The analysis remained rooted in participants’ accounts rather than decontextualized excerpts.

To support the trustworthiness of the findings, the researcher maintained a reflexive journal to document interpretive decisions, emotional reactions, and analytic shifts throughout the process. Three nursing professionals were also invited to read the interpretive stories. They included a current professor, a retired clinical nurse, and a retired nursing educator. Although they did not provide formal analytic commentary, their empathetic engagement affirmed the resonance and emotional truth of the narratives, while offering quiet support during emotionally difficult phases of the analysis.

As Frank [30] reminds us, dialogical research involves “being changed by what they say” (p. 966), and, as Ellis and Bochner [31] suggest, evocative narrative inquiry calls on researchers not only to interpret stories but to feel responsibly with them. These values shaped the analytic process from beginning to end.

## 3. Results

- Demographic Characteristics of Participants

Five female nurses, aged between 41 and 49, participated in this study. Four were married with one to three children, while one participant was divorced and living alone. All participants belonged to generation X, following the baby boomer generation, and were raised in blue-collar families.

They began their nursing education at the age of 15 through Taiwan’s junior college vocational track. Each nurse pursued further education while working, eventually completing a university degree. Notably, four of the five participants attained postgraduate qualifications or higher.

Their professional experience as registered nurses ranged from 21 to 30 years. Four of them remained loyal to their original hospitals throughout their careers and had never changed institutions. One participant had temporarily left nursing but ultimately returned to the profession and resumed work as a registered nurse.

- Thematic Findings

Through narrative thematic analysis, three overarching theme categories emerged, each encompassing several sub-themes that reflect participants’ lived experiences.

**Figure 1:**
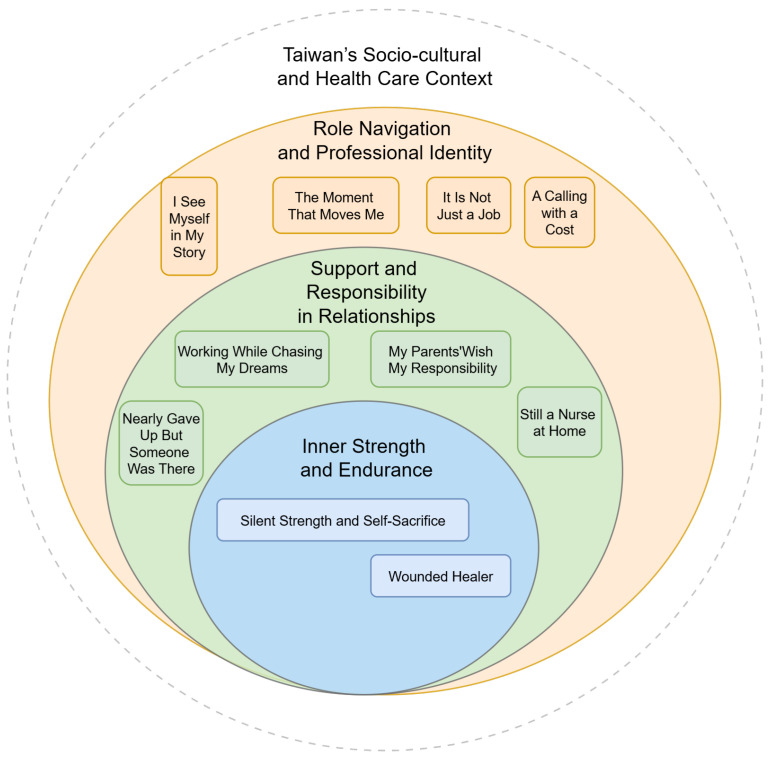
Concentric thematic map offering a visual overview of three narrative categories identified through thematic narrative analysis. From inner to outer rings: inner strength and endurance, support and responsibility in relationships, and role navigation and professional identity. Labels are rendered in first-person voice to foreground participants’ lived experiences. The dotted outer frame—Taiwan’s socio-cultural and health care context—symbolizes the cultural environment in which all narratives were situated.

To visually support the coherence and structure of these findings, a thematic map (Figure 1) was developed to illustrate the narrative categories and their interrelationship.

This figure is intended as a visual summary—not an analytic model or coding structure—but a synthesized landscape to support interpretive coherence and reader understanding. We hope this figure offers readers not only structural clarity, but a felt sense of the stories’ depth and interwoven meaning.

1. Inner strength and endurance
2. Support and responsibility in relationships
3. Role navigation and professional identity

These categories and their related sub-themes are summarized in Table 1.

### 3.1. Inner Strength and Endurance

This category highlights participants’ emotional resilience and capacity to persevere through both personal and professional challenges. Two sub-themes were identified: Silent strength and self-sacrifice and wounded healer.

#### 3.1.1. Silent Strength and Self-Sacrifice

Many participants described enduring personal struggles in silence—often to protect loved ones or avoid burdening others. This quiet resilience reflected a deep sense of responsibility and emotional restraint.

One participant shared a story about being hospitalized for surgery. While she was admitted, her mother came to the hospital’s outpatient clinic hoping to have lunch with her. She recalled the following:

“*I told her that I was busy that day and asked my friend to accompany her… After maybe half a year, when I was healthy again, I told my mom about the surgery. She said, ‘I knew something was unusual, but I couldn’t tell.’ She asked me not to keep quiet about this kind of thing again. But I just didn’t want her to worry.*”

(P05)

Another participant recounted an experience during early pregnancy. After noticing spotting, she rushed to the emergency room. Once the doctor confirmed she was fine to return to work, she did so the same day:

“*The doctor said I was okay to work, so I went on… You know we don’t have enough staff nurses. If I took leave, that would be a burden for others who would have to carry my duty.*”

(P02)

#### 3.1.2. Wounded Healer

Inspired by Carl Jung’s concept of the ‘wounded healer’ [32], this theme reflects how personal suffering became a source of empathy and transformation. Participants who experienced serious illness or emotional hardship described how these encounters deepened their approach to caregiving—allowing them to connect with patients with greater compassion and insight.

One nurse described being diagnosed with a tumor after a routine employee health check:

“*I was worried and felt uncertain… and then suddenly understood how patients feel… Now I’ve become more understanding and empathetic to my patients.*”

(P04)

Another participant, who faced a life-threatening illness, reflected the following:

“*I saw and witnessed many dying people and their families. Since I got very seriously sick, I reflected on my life… and this helps me understand and be able to discuss the topic of life and death with cancer patients… They knew I really understand.*”

(P05)

These stories revealed a quiet but powerful resilience. Participants often chose not to speak of their struggles not because they were unaffected, but because they carried a deep sense of duty to protect others. Whether facing personal illness or emotional strain, they persisted without complaint. Yet through those hardships, many found deeper empathy for patients and a renewed understanding of care. Enduring pain did not diminish their strength, it refined it.

### 3.2. Support and Responsibility in Relationships

This theme explores how interpersonal relationships and social expectations shaped participants’ career paths and personal growth. Four sub-themes were identified: My parents’ wish, my responsibility; nearly gave up, but someone was there; still a nurse at home; and working while chasing my dreams.

#### 3.2.1. My Parents’ Wish, My Responsibility

All participants began their nursing education at age 15, immediately after completing junior high school (Year 9), by entering a vocational track. Their decision to pursue nursing was largely shaped by their parents’ expectations—particularly among those from blue-collar backgrounds who viewed education as a path to stability and upward mobility. This theme reflects how familial aspirations and cultural values influenced their early career choices.

“*My parents were blue-collar working class. They didn’t have higher education, so they hoped I could have a better future. When I graduate, I can have a stable job for life.*”

(P01, P02, P04)

“*It’s because nursing can help others, and my father hoped I could be a nurse.*”

(P03, P05)

#### 3.2.2. Nearly Gave up, but Someone Was There

Several participants described moments of emotional distress and workplace bullying that pushed them to the brink of resignation or worse. In these critical moments, the presence of a supportive person, whether a friend, family member, faith community, or compassionate colleague played a pivotal role in helping them endure and recover.

One participant described being insulted by a physician who frequently teased her, saying things like, *“Why are you so stupid? Where is the data I asked for?”*

“*You know, that was very stressful, and sometimes I would just tear up… I didn’t know who I could share it with because he treated everyone like this… Then I went to church and asked my friends to pray for me… It made me pause and reflect, and I eventually resigned from that job. If my friends hadn’t supported me and prayed for me, I don’t know where I’d be.*”

(P04)

Two other participants reflected on having a difficult time with their nurse managers. One nearly resigned, while another considered jumping from a hospital window.

“*I was blessed that my former manager knew about my situation and helped me transfer to a new unit… Without her, I wouldn’t be here.*”

(P02, P05)

In describing her emotional distress, one participant added,

“*I was thinking about my family and my religion. I know they would be very sad if I really passed away… Without my family and my faith, I wouldn’t still be in this world.*”

(P02)

#### 3.2.3. Still a Nurse at Home

All participants described continuing their caregiving roles at home, especially when family members were ill. Their professional identity as nurses extended beyond the hospital, often leading to expectations from family and community that they would take charge of caregiving responsibilities. This theme also reflects cultural values around filial duty and the traditional role of women as primary caregivers.

“*I have no excuse for not taking care of others at home because they say, ‘You’re a professional.*”

(P05, P04)

“*I think I’m from a medical background and know how to communicate with other health professionals.*”

(P03)

“*I think it’s my duty to take care of seniors at home.*”

(P01, P02, P04, P05)

#### 3.2.4. Working While Chasing My Dreams

This theme highlights participants’ commitment to lifelong learning and professional growth. Despite the demands of full-time nursing work, several participants pursued higher education, completing undergraduate or graduate degrees while working. Their motivation stemmed from personal ambition, institutional expectations, and a desire to enhance their professional competence.

“*It was time for me to study. This is my duty and responsibility, and also a requirement of the hospital so I went to study.*”

(P01, P02, P05)

“*I realized I needed new knowledge for my work, so I went to study. I think I’m the most educated person in my family, it’s been a dream to pursue higher education.*”

(P04)

“*You know what? While working, I really enjoyed learning—not just in nursing, but from other professionals too. It helped me gain a broader vision and learn how to collaborate with others.*”

(P03)

Relationships, whether with family, faith, or trusted colleagues, played a vital role in how these nurses continued on their paths. For some, it was the weight of parental hope that launched their careers. For others, it was a conversation, a prayer, or a compassionate manager that kept them from giving up. These narratives show that nursing was not just a profession—it was shaped and sustained by the people around them. Even in moments of despair, connection pulled them through.

### 3.3. Role Navigation and Professional Identity

This category reflects how participants made sense of their roles and identities as nurses over time. Four sub-themes were identified: The moment that moves me; I see myself in my story; it is not just a job; and a calling with a cost.

#### 3.3.1. The Moment That Moves Me

Participants shared emotionally powerful moments that helped them realize the deeper meaning of their work. These experiences often involved profound human connection, witnessing healing, or being part of a patient’s journey. Such moments reinforced their sense of purpose and brought lasting job satisfaction.

“*When I was a new nurse, I thought working in the operating room was just a job you follow the routine. But then one day, we had an open-heart surgery on a baby. When the surgery was done, and all the tubes were removed, the moment the heart started to beat… I was very touched by that scene and realized, oh, this is meaningful.*”

(P03)

“*There was a young man the same age as me, but with the IQ of a 3-year-old. He needed help with daily activities like feeding and bathing. I remember one day, after we helped him bathe, he said, ‘That was so good.’ I felt so satisfied, knowing that even a small thing we did could comfort a patient.*”

(P05)

“*I think patients are our teachers. There was one patient who reminded me of my late father—he had a bad temper. But in the end, we became good friends. He called me ‘angel.’ The day before he passed away, he brought me a cup of coffee and had a heart-to-heart talk with me… He was one of the people who moved me and taught me about terminal care.*”

(P01)

#### 3.3.2. I See Myself in My Story

This theme emerged from participants’ reflections after sharing their personal narratives. The act of telling their stories, especially those involving pain, injustice, or long-held silence, became a moment of healing and self-discovery. Participants described feeling seen, validated, and reconnected with their professional identity and purpose.

“*I was healing and gaining understanding while I shared my story about the physician insulting me. For such a long time, I kept this to myself and didn’t expect others to understand. I lost my confidence… but after talking about it, I knew I didn’t do anything wrong. There is a purpose for this experience. I saw myself again through telling it all out.*”

(P04)

“*This review helped me reflect on my journey, and I re-met myself again… I felt awakened and ready to do something right to improve nursing.*”

(P01)

“*It’s been almost 30 years since I started nursing. So many things have happened in my life. I felt amazed, and my original intention of becoming a nurse came back to me.*”

(P05)

#### 3.3.3. It Is Not Just a Job

Participants emphasized that nursing is more than just a job—it is a meaningful and deeply personal calling. Despite the challenges, they expressed pride, love, and a strong sense of purpose in their work.

“*Nursing is beautiful. I still love it. It’s not easy, especially now with the shortage of staff. Nurses, rise up! We need to support ourselves.*”

(P01, P02, P05)

“*Nursing can help others and our families. You may find it very useful and meaningful in many ways.*”

(P03)

“*I hope I can be a vessel for God through my role as a nurse.*”

(P04)

#### 3.3.4. A Calling with a Cost

While participants found nursing to be meaningful and fulfilling, some expressed hesitation about encouraging their children to enter the profession. They cited emotional strain, physical exhaustion, and system-level challenges as reasons for concern. This tension reveals the complexity of professional identity: pride in the role coexists with fears about its sustainability and long-term impact on personal well-being.

“*I don’t think I will encourage my daughter to be a nurse unless we, as a profession, make some changes.*”

(P01)

“*I won’t ask my children to enter this profession. Even though I love nursing, as you know—it’s just too hard and tough.*”

(P05)

These stories show how identity is not something fixed, but something participants continuously revisited and reshaped. Through meaningful moments at the bedside, or in reflection on past struggles, they rediscovered why they chose nursing in the first place. Telling their stories reminded them of their strength, their compassion, and their purpose. Even as they questioned the cost of their calling, they spoke with pride and with hope that nursing can become something better for those who follow.

Together, these themes illustrate the deeply personal, relational, and ethical dimensions of nursing as experienced by senior staff nurses in Taiwan. From silent strength to transformative encounters, the participants’ narratives reveal how professional identity is shaped not just by clinical practice, but by a lifetime of caregiving, personal growth, and social influence. The following section will further explore the meaning and implications of these themes in light of existing literature and cultural context.

## 4. Discussion

This study explored how senior nurses in Taiwan make sense of their professional lives—not only through what they do, but through who they are becoming. As [20] remind us, professional identity is always shaped by time, place, and relationships. In this study, participants’ stories showed that nursing identity is not built only through clinical practice but also through cultural values like family duty, gender roles, and shared caregiving. Some talked about staying silent to protect others, carrying pain alone, and rediscovering parts of themselves while telling stories they had never shared before. These moments were not dramatic, but they were deeply human. Professional identity, in this sense, is not something fixed or achieved, it is something lived and reshaped again and again through care and connection. In a culture where duty and relationship matter deeply, staying in nursing is rarely about the system. It is about meaning, presence, and the quiet strength of continuing on.

All five participants in this study were women, reflecting the reality that over 95% of Taiwan’s nurses are female [33]. Globally, women also make up about two-thirds of the health and social care workforce [34]. This gender imbalance is often accepted as fact, but it also reflects deeper cultural beliefs about who is expected to care. In Taiwan, nursing often continues the idea that women should be caregivers—not just at work, but at home. It is more than a job. It is an extension of what daughters and wives are expected to be.

Most participants did not choose nursing on their own. Instead, their families guided them toward it, often saying it was a safe or respectable path. In many cases, career choice was not personal, but a way to fulfill hopes for future stability and family pride. This reflects the idea of authoritarian filial piety [35], where daughters are expected to put family needs before their own. National data support this: Nearly half of junior college nursing students in Taiwan chose the field because of family expectations [36], but many also expressed doubt or a wish to leave. This shows a quiet struggle between respect and resistance. Some carry their choice with pride; others carry it with silence. Either way, the early decision to enter nursing shapes how identity is formed over time.

For these nurses, cultural and professional expectations often blended together. Confucian values like harmony and duty were matched by a nursing culture that rewards self-sacrifice. Many participants said they became nurses because others suggested it, not because they felt called. Over time, they developed strong ethical commitments. However, their stories also revealed a pattern of putting others first without question. One returned to work on the day she had pregnancy bleeding; another hid her own hospitalization from her family. These were not seen as choices, but as part of being who they were a nurse, a caregiver, a daughter. When giving becomes a duty instead of a choice, as Pask [37] warns, it can slowly wear away a person’s sense of self.

Participants also spoke about caregiving at home. Their nursing skills often made them the default caregiver for elderly parents or sick relatives. However, this led to extra responsibility—emotional and physical—that few others could fully share. It echoes findings from Kokorelias, et al. [38] that healthcare professionals who also provide care at home often feel stretched and unsupported. Local research in Taiwan further affirms the structural roots of this dual caregiving burden. Chang [39] demonstrates that female healthcare personnel—especially nurses—not only perform emotional labor within hospitals but also shoulder unpaid family caregiving responsibilities at home. These expectations are culturally framed and institutionally maintained, contributing to invisible labor and long-term exhaustion. For many in this study, the pressure to give was constant, at work and at home, with little room to rest.

All participants were born between the late 1970s and early 1980s a generation shaped by Taiwan’s shift into the digital age. As students, they were among the first to use computers; as professionals, they adapted to electronic records and new technologies in care. They often had to figure things out on their own. While lifelong learning is a well-known part of professional growth [40,41], for these nurses, it was not just a goal it was a way to survive. Many returned to school mid-career while juggling full-time work and family care. In their stories, education was not ambition—it was persistence. Like findings by Tussing, et al. [42] suggest, generation X nurses are quietly adaptable and self-motivated. For these nurses, further study was more than a step up, it was a form of reclaiming identity in systems where their value had been long overlooked.

This study offers a nuanced contribution to Taiwanese nursing scholarship by illuminating how long-serving nurses sustain meaning and commitment over time—not through institutional incentives or promotion, but through emotionally resonant encounters, relational ethics, and culturally embedded caregiving. While previous research has explored burnout, retention, and role strain, few studies have examined vocational identity formation across decades of clinical practice.

By using first-person thematic mapping and narrative inquiry, this study highlights dimensions of nursing that are often absent from quantitative or policy-driven analyses—such as emotional truth, ethical ambiguity, and the co-construction of self in caregiving relationships. These insights deepen local nursing discourse and offer interpretive clarity about what it means to remain a nurse across generational, cultural, and relational thresholds.

In their stories, resilience came not from being tough alone, but from staying connected. They stayed not because systems helped them but because people needed them. A look from a patient, a kind colleague, or a word of encouragement was often enough. This reflects the idea of relational resilience [43], or the strength that is built in relationship. However, it also shows a problem. When nurses are always expected to stay strong for others, and systems offer little in return, that care becomes heavy. As Buchan and Catton [44] caution, asking nurses to remain resilient without addressing the structural conditions that exhaust them risks transforming care into a burden rather than a vocation. In these stories, we do not just hear endurance, we also hear a quiet wish to be cared for. Although the study took place during the COVID-19 period, participants rarely voiced the pandemic directly. Instead, they spoke of histories, relationships, and enduring meanings that stretched far beyond any single moment of crisis. What is absent from a story may carry weight equal to what is spoken. In narrative inquiry, silence is not lack—it is choice. It is shaped by what matters most, what lingers, and what resists being named.

Although participants rarely used the term “well-being,” their stories spoke quietly of it. Through relationships, reflection, and moments of meaning, they found ways to keep going—even when systems offered little support. These forms of strength were not always visible, but they were sustained over time. In this way, narrative inquiry became more than a method of data collection—it created a space for reconnection, for naming what had long been carried alone, and for glimpsing a form of well-being shaped by care, culture, and endurance.

While participants shared many moments of pain, struggle, and sacrifice, their stories also became places of reflection and healing. As Holloway and Freshwater [45] remind us, narrative research is not only a method—it is a way to reconnect with meaning. Some participants said they felt seen or that they rediscovered parts of themselves through telling their stories. Narrative, in this sense, became more than a tool for research. It became an act of healing. Through their words, nurses moved from silent endurance to reflective authorship. Their stories were not just reports of what happened, but acts of reclaiming who they are. In that way, narrative was not only a method. It was an ethical gesture—one that returned voice and dignity to those often unheard.

## 5. Limitations

This study began with a commitment to narrative inquiry—not only as a methodology, but as a way of being, thinking, and listening to lived experience. While this approach enabled deep engagement with participants’ life stories, several limitations remain.

First, the participant group included five senior staff nurses born in the 1970s to early 1980s, all of whom identified as female and had sustained clinical practice in Taiwan. Their narratives are situated within Taiwan’s culturally embedded healthcare landscape, shaped by Confucian ethics, generational values, and gendered expectations. As such, findings may not be transferable to male nurses, younger cohorts, or those in non-clinical or administrative roles.

Second, the sample size—though intentionally small—limits generalizability. Narrative inquiry emphasizes meaning-making through sustained dialogue and relational co-construction, rather than statistical representation. As Dahal et al. [46] note, qualitative studies using narrative methods often include as few as one to six participants when prolonged story generation is central to the design. The deliberate emphasis on depth over breadth supported this study’s exploration of professional identity, caregiving ethics, and long-term endurance.

Third, while reflexive journaling and member checking were conducted to enhance trustworthiness, the narrative accounts are co-constructions between participant and researcher. These stories are situated voices—not objective truths—and are offered not for replication, but for resonance. The truths they carry are not universal—but they are deeply lived, and quietly powerful.

Lastly, the pandemic’s narrative absence speaks its own truth. Although interviews spanned the COVID-19 period, participants rarely voiced it directly. Their choice to foreground long-term caregiving, relational endurance, and personal resilience may reflect deeper ontological priorities. Silence, in this context, was not an omission but a meaningful withholding—what lingered most was not the crisis, but the stories that outlasted it.

## 6. Implications for Nursing Practice, Education, and Research

Based on the storied experiences of senior nurses, this study highlights the importance of sustaining professional identity across relational, cultural, and emotional dimensions. Narrative inquiry facilitates deeper understanding in clinical education and mentoring, especially regarding ethical endurance and gendered caregiving. Future nursing research may benefit from participatory designs that honor lived experience and context-sensitive storytelling.

These findings offer layered insight into how senior staff nurses sustain professional identity, relational caregiving, and ethical endurance over time. Their stories invite reflection on how healthcare systems recognize—not merely retain—experienced nurses. This ethical stance aligns with the principle of lifelong learning, as articulated in the 2023 Taiwan Code of Ethics for Nurses [41], which states that “Nurses engage in continuous professional development and lifelong learning to enhance professional competence and fulfill the professional responsibilities of nursing practice.”

Through mentoring, educational pursuit, and relational endurance, the senior nurses in this study embody lifelong learning not as a requirement, but as a vocational ethic. Their stories reveal that continued growth is both an act of care and a quiet commitment to sustaining the professional spirit of nursing across generations.

Local sociological research also affirms the structural roots of these burdens. Chang [39] demonstrates that female healthcare personnel—especially nurses—not only perform emotional labor within hospitals but also shoulder unpaid caregiving responsibilities at home. These expectations are culturally framed and institutionally maintained, contributing to invisible labor and long-term exhaustion.

In clinical practice, the narratives encourage more intentional support for long-serving nurses as moral anchors, informal mentors, and relational caregivers within teams. Institutions might consider programs that foster intergenerational exchange, protect the quiet strength of caregiving, and honor lived experience as a form of leadership.

In nursing education, narrative inquiry becomes more than a pedagogical tool—it is a developmental space for professional identity. As Benner [47] emphasizes, experiences of first-person narratives help capture clinical reasoning, perceptual skill, and ethical responsiveness within dynamic contexts. These narratives do not simply describe events; they allow nurses to reflect on “knowing how and when,” to make sense of what mattered, why, and who they were in moments of care. In this view, storytelling fosters not only knowing, but becoming—supporting learners in articulating professional identities through lived complexity.

For research, this study affirms narrative inquiry as a rigorous and relational methodology. Future work may explore how identity is shaped across shifting cultural, technological, and post-pandemic terrains, and how silence, care, and endurance are ethically narrated across roles, generations, and genders.

## 7. Conclusions

This study gave voice to the lived experiences of senior nurses in Taiwan, women who have carried both visible and invisible forms of care through decades of professional and personal responsibility. Their stories showed how nursing identity is not fixed, but shaped and reshaped through cultural values, relationships, and reflection. These narratives remind us that nursing is not only a job; it is a way of being, held together by meaning, silence, and quiet strength.

As a narrative study, the goal was not to generalize or represent all nurses, but to understand how identity is lived and made sense of in context. While the small, specific sample may limit transferability, it also allowed for depth, trust, and ethical closeness between researcher and participant. Future studies may explore different generational or regional perspectives to continue this conversation.

At the same time, these stories offer meaningful implications. For nursing practice, they show the importance of recognizing not just what nurses do, but who they are and how they are shaped by cultural and relational forces. For education, they suggest that narrative can be more than a method of research; it can also be a practice of self-reflection. Creating space for nurses to tell, revisit, and reframe their own stories may help renew professional identity and well-being. In systems that often overlook emotional needs, listening to story becomes an act of care.

What these nurses shared is not meant to speak for everyone. But each story carried something quietly true. To care for those who care, we must make room for their voices. Not just in research but in practice, education, and leadership. Their stories are not the end of something, but a beginning.

## Figures and Tables

**Table 1:** Summary of thematic categories and sub-themes.

Theme Category	Sub-Theme	Description
Inner strength and endurance	Silent strength and self-sacrifice	Quietly enduring personal hardships to protect others or avoid burdening colleagues.
	Wounded healer	Transforming personal suffering into empathy and deeper care for patients.
Support and responsibility in relationships	My parents’ wish, my responsibility	Early nursing career shaped by family expectations and socio-economic values.
	Nearly gave up, but someone was there Still a nurse at home Working while chasing my dreams	Support from others sustained participants during emotional distress and workplace adversity. Continued caregiving responsibilities within the family, reflecting cultural and gendered expectations. Pursuing higher education while working, driven by ambition, institutional demands, and love of learning.
Role navigation and professional identity	The moment That moves me	Emotional and meaningful patient interactions that reaffirmed professional purpose.
	I see myself in my story	Healing and self-recognition through the act of storytelling.
	It is not just a job A calling with a cost	Nursing is perceived as a vocation—a meaningful and enduring calling beyond duty. Ambivalence toward recommending nursing to the next generation due to emotional and physical toll.

Sub-themes were developed through interpretive analysis of participants’ stories across personal, relational, and professional domains.

## Data Availability

Due to the narrative and confidential nature of the data, the interview transcripts are not publicly available. Anonymized thematic descriptions may be provided upon reasonable request.
